# Analysis of pH, calcium ion release, and energy dispersive spectroscopy of a bioceramic root canal dressing

**DOI:** 10.1590/0103-6440202305506

**Published:** 2023-10-27

**Authors:** Bárbara Luzia Capitanio, Lina Naomi Hashizume, Milton Carlos Kuga, Eliane Cristina Gulin de Oliveira, Ricardo Abreu da Rosa, Gabriel Barcelos Só, Marcus Vinicius Reis Só

**Affiliations:** 1 School of Dentistry, Department of Conservative Dentistry, Federal University of Rio Grande do Sul - UFRGS, Porto Alegre, Brazil.; 2 School of Dentistry, Department of Restorative Dentistry - UNESP, São Paulo State University, Araraquara, Brazil.

**Keywords:** calcium silicate-based medication, calcium hydroxide-based medication, energy dispersive X-ray spectroscopy, pH, calcium ions release

## Abstract

This study compared the pH and calcium ion release of calcium silicate- (Bio-C Temp) and calcium hydroxide-based (Ultracal XS) medications. Intracanal remnants of both medications were also evaluated using SEM-EDS after the removal protocol. Thirty-five bovine teeth were prepared. Fifteen were filled with Bio-C Temp and 15 with Ultracal XS. Five remained without intracanal medication (control group). Five samples from each experimental time (i.e.. 24, 72, and 168 hours) were used to measure pH and calcium ions release using a digital pH meter and microplate reader, respectively. Afterward, the peaks of the chemical elements composing both medications were analyzed in SEM-EDS. One-way ANOVA and Tukey's post hoc test analyzed the pH and calcium ion release data. Student's t-test compared the medications in each experimental time. SEM-EDS described the percentage of chemical elements in the samples. Bio-C Temp and Ultracal XS showed a significant pH increase from 24 to 168 hours (p<0.05). Ultracal XS showed a higher pH value at 24 hours than Bio-C Temp (p<0.05) but were similar at 72 and 168h (p > 0.05). Calcium ion release did not depend on the experimental period (p > 0.05). Bio-C Temp showed lower calcium ions release than Ultracal XS at 24 hours (p<0.05). SEM-EDS analyses showed the remains of both medications, but the concentration of Si, Al, and W ions was present only in the calcium silicate-based medication. Bio-C Temp presented alkaline pH and a satisfactory calcium ion release over the time. The remaining of both medications were present after the protocols for paste removal.

## Resumo

Este estudo comparou o pH e a liberação de íons cálcio de medicações intracanais a à base de silicato de cálcio (Bio-C Temp) e à base de hidróxido de cálcio (Ultracal XS). Remanescentes de ambas as medicações também foram avaliados usando microscopia eletrônica de varredura e espectroscopia de dispersão de energia após o protocolo de remoção. Trinta e cinco dentes bovinos foram preparados. Quinze dentes foram preenchidos com Bio-C Temp e 15 com Ultracal XS. Cinco permaneceram sem medicação intracanal (grupo controle). Cinco amostras de cada tempo experimental (ou seja, 24, 72 e 168 horas) foram usadas para medir o pH e a liberação de íons de cálcio usando um medidor de pH digital e um leitor de microplacas, respectivamente. Em seguida, os picos dos elementos químicos que compõem os dois medicamentos foram analisados ​​em microscopia eletrônica de varredura e por espectroscopia de dispersão de energia. Os testes One-way ANOVA e post hoc de Tukey analisaram os dados de pH e liberação de íons cálcio. O teste t de Student comparou as medicações em cada tempo experimental. A microscopia eletrônica de varredura e a espectroscopia de dispersão de energia descreveu a porcentagem de elementos químicos nas amostras. O Bio-C Temp e o Ultracal XS mostraram um aumento significativo de pH de 24 a 168 horas (p<0,05). O Ultracal XS apresentou um valor de pH mais alto em 24 horas do que o Bio-C Temp (p<0,05), mas foi semelhante em 72 e 168h (p > 0,05). A liberação de íons cálcio não dependeu do período experimental (p> 0,05). O Bio-C Temp apresentou menor liberação de íons de cálcio do que Ultracal XS em 24 horas (p<0,05). As análises de microscopia eletrônica de varredura e espectroscopia de dispersão de energia mostraram remanescentes de ambas as medicações, mas a concentração de íons Si, Al e W estavam presentes apenas na medicação à base de silicato de cálcio. O Bio-C Temp apresentou pH alcalino e maior liberação de íons cálcio. Remanescentes de ambas as medicações estiveram presentes após os protocolos de remoção da pasta.

## Introduction

Bacteria and their by-products play an essential role in initiating and perpetuating pulpal and periapical pathologies ^1^. The reduction of these infectious agents in the root canal is performed using mechanical preparation of the root canal system associated with irrigating solutions and antibacterial agents, such as intracanal medications ^2^ ^,^ ^3^. Areas of anatomical complexity hamper the action of instruments and irrigants during endodontic treatment, and even in well-conducted endodontic treatments, the infection can persist ^4^. It is possible to optimize the disinfection using an intracanal medication ^5^.

Calcium hydroxide paste is the most recommended intracanal medication due to its physical, chemical, and biological properties. It has an antibacterial effect ^6^, highly alkaline pH, anti-inflammatory capacity, and ability to dissolve organic tissue and induce repair by deposition of mineralized tissue ^7^.

More recently, a calcium silicate-based medication has been developed and tested (8-13). Bio-C Temp (Angelus Indústria de Produtos Odontológicos S/A; Londrina, Brazil) is an intracanal medication composed of glycol salicylate ester, titanium oxide, calcium aluminate, calcium oxide, calcium tungsten, and calcium silicate. Recent studies present this medication as a material with high calcium release ^8^ ^,^ ^10^, alkaline pH ^8^ ^,^ ^10^ ^,^ ^12^, acceptable radiopacity ^8^ ^,^ ^9^, and biocompatibility ^9^ ^,^ ^13^. Compared with calcium hydroxide-based medications, it had less antibacterial capacity ^11^ ^,^ ^12^ but similar cytocompatibility and induction of mineralized tissue ^11^.

It has been demonstrated that Ca(OH)2 dressing remnants influence the penetration of root canal sealers into dentinal tubules ^14^ and may compromise endodontic sealing ^15^. So far, there is no evidence regarding residual bioceramic medication (Bio-C Temp) on the dentin wall and its impact on the obturation of the root canal system. Recently, the study developed by Kegler ^16^ evaluated the influence of residues from the intracanal medications Bio-C Temp and Ultracal XS on bond strength and adhesive interface formation of epoxy resin-based and bioceramic-based root canal sealers. The author showed that residues of Bio-C Temp improved the adaptation of a calcium silicate-based endodontic sealer.

Therefore, this study aimed to compare the pH and the calcium ions released from a calcium silicate- (Bio-C Temp) and a calcium hydroxide-based (Ultracal XS) root canal dressing. It also aimed to evaluate the presence of intracanal remnants of both medications using SEM-EDS. The null hypothesis was that the materials tested would present similar properties at pH and calcium release evaluations.

## Materials and methods

The Federal University of Rio Grande do Sul Research Ethics Committee approved this research, process number 4.948.911.

### Sample calculation

For the sample calculation, the T-test was performed using the statistical package BioEstat 5.0 (Fundação Mamirauá, Belém, Pará, Brazil), considering a confidence interval of 95% and power of the statistical test of 80%, based on the studies of Dudeja et al. ^17^ and Zmener, Pameijer and Banegas ^18^ for calcium release and pH analysis, respectively.

Furthermore, RoBDEMAT (A risk of bias tool and guideline to support reporting of pre-clinical dental materials research and assessment of systematic reviews) ^19^ was used to assess and evaluate the quality of this*in vitro*study concerning dental materials research.

### Obtaining and preparing the samples

Thirty-five bovine teeth were selected. It excluded teeth with root lengths lower than 15 mm, without root fractures or cracks detected through a visual examination, and without internal or external resorptions verified by previous radiographs obtained with a digital system (Gnatus, São Paulo, Brazil), and immature roots.

After selection, the teeth were stored in flasks with 5% sodium hypochlorite for 24 hours and then in flasks with phosphate buffer (PBS). All specimens were radiographed buccolingually and mesiodistally before preparation. Then, the roots were sectioned with a diamond disk, so they all were 12 mm long.

Root canal negotiation was performed with a K-file #15 (Dentsply-Maillefer, Ballaigues, Switzerland) and irrigation with 5 mL of 2.5% sodium hypochlorite. A K-file #15 was inserted into the root canal until its tip coincided with the foramen. The working length was established as 1 mm shorter than this measure. The canal preparation was performed with Wave One Gold Large 45.05 instruments (Dentsply Maillefer, Ballaigues, Switzerland) up to the working length using a VDW Silver electric motor (VDW Company, Munich, Germany). Next, the canals were manually prepared up to a K-file #60 on the working length.

During preparation, irrigation was performed with 10mL of 2.5% sodium hypochlorite. Afterward, the following protocol for final irrigation was performed: passive ultrasonic irrigation (PUI) with 5mL of 2.5% sodium hypochlorite for 1 minute, interspersed in 3 periods of 20 seconds ^15^. During the first two periods, 2mL of NaOCl was delivered, and, in the third period, 1mL. Afterward, irrigation was performed with 5mL of saline solution. Finally, final irrigation with 2 mL of 17% EDTA (ASFER, São Caetano do Sul-SP) and 30 seconds of PUI was performed. Five milliliters of saline solution removed the EDTA from the canals. All irrigation procedures were performed with disposable syringes (Ultradent Products Inc., USA) and Endo-EzeTip needles (Ultradent Products, USA). PUI was always performed using an Irrissonic tip (Irrisonic; Helse Ultrasonics, SP, Brazil) and an ultrasonic unit at 20% power, coupled to the handpiece of the ultrasound device (Newtron P5 xs - Acteon - Satelec, Indaiatuba, SP, Brazil) ^20^.

In the Bio-C Temp group (calcium silicate-based medication-test group), an endodontic tip only aspired the excess of saline solution from the canals to keep the dentin walls slightly wet. In the Ultracal XS group (calcium hydroxide-based medication- positive control group), the canals were dried using absorbent #60 paper cones (Dentsply-Maillefer, Ballaigues, Switzerland). Fifteen teeth were filled with Ultracal XS and fifteen with Bio-C Temp. Both were inserted with a NaviTip needle (Ultradent Products, USA) until the medication was visible at the root canal entrance. New radiographs were taken to verify the correct filling of the canals. Then, all teeth were restored with glass ionomer cement (Riva Light Cure - SDI Limited, Victoria, Australia).

All specimens were individually stored in plastic flasks with 10 mL of deionized water at 37°C, five specimens per group for 24 hours, five for 72 hours, and the other five for 168 hours. Five teeth in the control group were prepared and stored in deionized water for 168 hours but remained without intracanal medication.

### pH measurement and determination of calcium ion release

###  pH measurement 

At each experimental time (24h, 72h, and 168 hours), the pH value of the solutions where the samples were immersed was measured.

In this analysis, five specimens for each experimental time of each group (Bio-C Temp and Ultracal XS) were evaluated. Before the readings, the teeth were removed from the flasks, and the solutions were manually agitated for 5 seconds. The pH was measured with a digital pH meter (Digimed DM-22, São Paulo, SP, Brazil) previously calibrated with solutions with known pH. This method was controlled by reading the pH values of deionized water in which no sample was immersed.

###  Determination of calcium ion release 

Calcium ion release was evaluated at the same experimental periods used for pH analysis (24, 72, and 168 hours). This analysis used a colorimetric method employing the chemical reagent Arsenazo III. After that, the reading was performed in a microplate reader (Multiskan GO, Thermo Fisher Scientific, USA) at a 650nm wavelength.

After evaluation at each experimental time, the samples were immersed in PBS solution until the removal of the medications.

### Protocol to remove the intracanal dressing

After pH and calcium ion release procedures, the pastes were removed from the root canals. PUI was always performed using an Irrissonic tip (Irrisonic; Helse Ultrasonics, SP, Brazil) and an ultrasonic unit at 20% power, coupled to the handpiece of the ultrasound device (Newtron P5 xs - Acteon - Satelec, Indaiatuba, SP, Brazil) ^20^

The canals were negotiated again with a K-file #60, irrigated with 2.5% NaOCl, and three cycles of 20 seconds of PUI were performed. 5 mL of NaOCl was used 2 mL in the first 20 seconds of activation, 2 mL for the second, and 1 mL for the third activation ^20^. Afterward, the canals were irrigated with 5 mL of saline solution. PUI was performed as described before.

### Scanning electron microscopy/energy dispersive X-ray spectroscopy (SEM/EDS) analysis

The roots selected for SEM/EDS analysis were longitudinally sectioned. The roots were grooved in a bucco-lingual direction without penetrating the root canal. A chisel (# 1L, SSWhite/Duflex, Rio de Janeiro, RJ, Brazil) was placed in the groove to split the root into two halves with gentle pressure. The sections were dried at room temperature, dehydrated in a dissector with silica gel for one week, and fixed on aluminum stubs. Then, they were sputter-coated with carbon (Bal-Tec, Balzers, Liechtenstein).

Six specimens from each group (Bio-C Temp and UltraCal XS) and two specimens from the negative control group were analyzed using scanning electron microscopy with JEOL 6060 (JEOL 6060; JEOL, Ltd., Tokyo, Japan) and submitted to carbon coverage (BalTec SCD 004 COater; Balzers, Vaduz, LI), at 15 kV for 180 seconds. Through SEM, it was possible to observe if there were remnants of medication on the dentin surface. Then, to verify the peaks of the chemical elements, the dentin surface was submitted to EDS (JEOL 6060; JEOL, Ltda Tokyo, Japan), evaluating the percentage peaks of the chemical elements found in the sample. Four fields (two in the middle and two in the apical third) were evaluated. The most representative image, chosen at the interface between the root canal wall and residual material, was obtained at 500x magnification. Only one experienced operator obtained all the images.

### Statistical analysis

The pH and calcium ion release data were analyzed by one-way ANOVA and Tukey's post hoc test. A student's t-test compared Ultracal XS and Bio-C Temp at each experimental time. The significance level was set at 5%. The SEM/EDS analysis describes the concentration percentage of chemical elements per group. BioEstat 5.3 (Instituto Mamirauá, AM, Brazil) software was used.

## Results

### pH measurement

Bio-C Temp significantly increased from 24 to 168 hours (p < 0.05). Ultracal XS has higher pH values at 72 and 168 hours (p < 0.05). But without differences between both experimental times (p > 0.05) (Table 1).

**Table 1:** pH values of intracanal medications at different experimental times.

pH	24h	72h	168h
Bio-C Temp	7.20 ± 0.15 Cb	8.00 ± 0.24 Ba	9.04 ± 0.28 Aa
Ultracal XS	7.84 ± 0.08 Ba	8.40 ± 0.79 Aa	8.78 ± 0.27 Aa
Calcium			
Bio-C Temp	208.07 ± 48.30 Ab	262.71 ± 97.71 Aa	287.86 ± 84.26 Aa
Ultracal XS	315.89 ± 76.50 Aa	327.05 ± 73.64 Aa	330.59 ± 96.96 Aa

Capital letters compare experimental times according to each medication (in the row)

after one-way ANOVA and Tukey's post hoc tests.

Small letters compare both medications at each experimental time (in the column) after student’s t-test.

When both medications were compared at each experimental time, significant differences were found only in 24 hours (p < 0.05). Bio-C Temp showed lower pH values in 24h than Ultracal XS (p < 0.05) (Table 1).

### Calcium ion release

When both medications were compared within each experimental time, Bio-C Temp showed lower calcium ion release at 24 hours than Ultracal XS (p < 0.05). At 72 and 168 hours, the calcium ions release was similar for both medications (p > 0.05) (Table 1).

### SEM/EDS analysis

Remnants of both medications were observed in SEM/EDS analysis. However, the Si, Al, and W ions concentration was present only in Bio-C Temp since these ions are part of the medication formulation. The Ca/P ratio is slightly increased for both Ultracal XS and Bio-C Temp compared to the control group (Table 2).

**Table 2:** Mean and standard deviation (SD) of ions concentration (in percentage), in relation to the total amount of chemical elements detected by EDS.

		Bio-C Temp	Ultracal XS	Control
Calcium/Phosphore (Ca/P)	Mean	2.24	2.37	2.05
	SD	0.02	0.30	0.08
Calcium (Ca)	Mean	15.85	14.37	15.89
	SD	1.93	1.52	1.97
Silicon (Si)	Mean	0.51	0	0
	SD	0.19	0	0
Aluminum (Al)	Mean	0.16	0	0
	SD	0.13	0	0
Tungsten (W)	Mean	1.82	0	0
	SD	2.14	0	0

**Figure 1:**
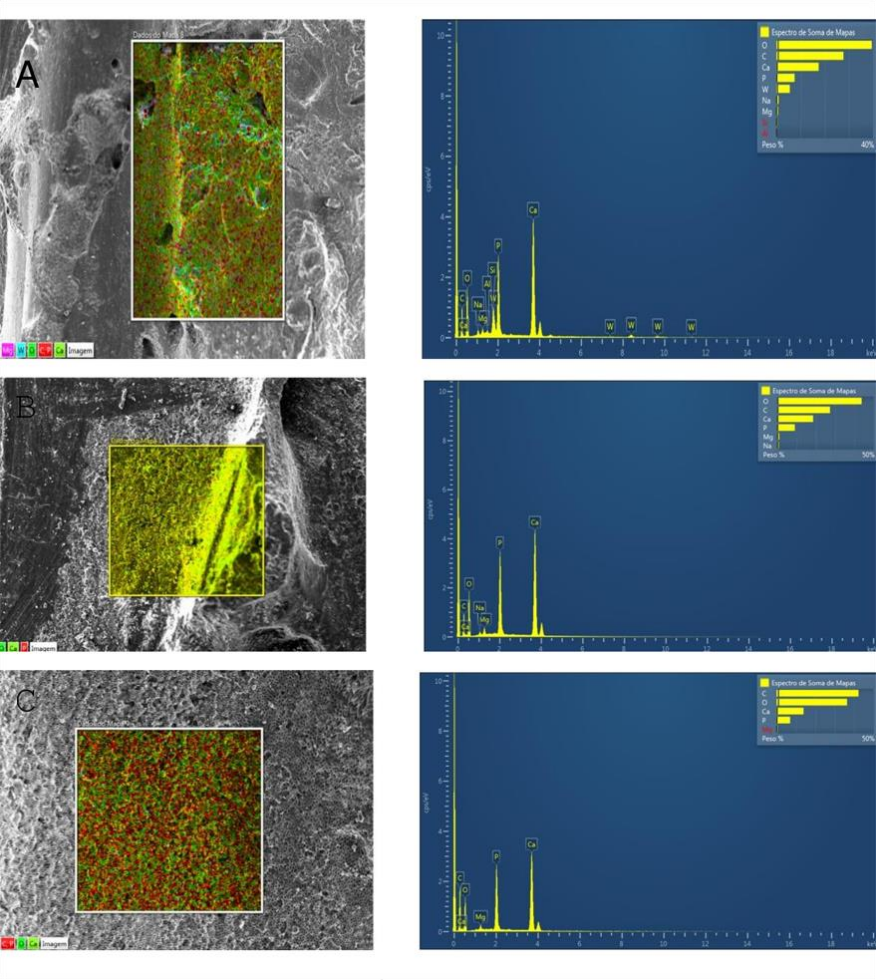
Representative SEM/EDS image of the dentin surface after removal of Bio-C Temp (A), Ultra Cal XS (B), and control group (C); 500µm scale

Figure 1 presents illustrative SEM/EDS images of the dentin surface after removing Bio-C Temp, UltraCal XS, and the negative control group. It is possible to observe the image of the dentin wall with medication remnants and the peaks of the chemical elements that constitute the materials (A, B). C represents the control group, where none of the medications were used.

## Discussion

Both medications had an alkaline pH, which favors the elimination of microorganisms involved in endodontic infections ^21^. An alkaline pH (8.6 to 10.3) is necessary to obtain the biological action of intracanal medications ^22^. However, a pH above 11 can be cytotoxic to periapical tissues ^23^. Therefore, according to this study, both medications had pH within acceptable limits, reaching the necessary alkalinity in the experimental time of 168 hours. In the intra-group analysis, Bio-C Temp showed a significant increase in pH values throughout the experimental period, corroborating another study ^8^. Ultracal XS presented a significantly more alkaline pH than Bio-C Temp only in the 24 hours. Other studies found higher values for calcium hydroxide-based pastes in all experimental times ^8^ ^,^ ^10^ ^,^ ^12^. Methodological differences can explain this result. The cited studies immersed polyethylene tubes filled with the studied medications in deionized water. Conditions closer to the oral environment were reproduced in the present study since the medications were inserted inside the root canals, leaving only the apical foramen orifice without sealing.

Bio C Temp and Ultra Cal XS showed a gradual increase in calcium ion release throughout the experimental period. However, significant differences occurred only between 24, 72 and 168 hours for Bio-C Temp. Previous studies also found this result ^8^ ^,^ ^10^. The ability to release calcium ions is an important property, as it can favor alkaline pH ^24^ and contributes to the stimulation of repair by depositing mineralized tissue. In addition, it reacts with carbon dioxide, affecting the survival of anaerobic bacteria present in endodontic infections ^25^.

SEM/EDS analysis allowed for delimiting the dentin surfaces impregnated by the tested medications and confirming their presence by quantifying the concentration of chemical elements in the area. The EDS analysis showed the presence of calcium, phosphate, silica, aluminum, and tungsten in the Bio-C Temp group. All these chemical elements are present in the composition of the bioceramic paste. In the control group, which remained without intracanal medication, calcium, and phosphate, elements in the dentin composition were indicated.

In both experimental groups, intracanal medication residues were found on the dentinal walls, corroborating the previous literature ^26^ ^,^ ^27^. These residues can subsequently interfere with the adhesion of endodontic sealers to the walls of the root canals and their penetration into the dentinal tubules, influencing the quality of the endodontic filling ^28^ ^,^ ^29^. Kegler ^16^ showed a biomineralized layer formed from a chemical interaction between the dentinal wall, the medication, and the bioceramic sealer.

The chemical composition of this mineralized interface possibly comes from the calcium and hydroxyl ions released by the intracanal medication, which interact with calcium silicates, oxides, and aluminates in the sealer, generating calcium phosphate, calcium carbonate, hydroxyapatite, and carbonated apatite ^30^. Although it was not the target of the present study, this interaction and its long-term biological and mechanical behavior still need to be completely elucidated. On the other hand, when using calcium hydroxide-based medication, the study by Kegler ^16^ reported lower bond strength and a higher percentage of failures at the sealer/dentin interface. The presence of residual calcium hydroxide-based medication can interfere with the physicochemical properties of sealers and act as a physical barrier between the filling material and the dentin surface ^29^.

This study could observe some limitations, such as other analyses: antibacterial activity, radiopacity, dentinal tubule penetration, and root canal dressing removal. These experimental tests could motivate other studies with this new bioceramic root canal dressing.

The clinical relevance of this study rests on the increasing use of calcium silicate-based materials in Endodontics, and the use of a bioceramic intracanal medication appears as another biological alternative.

## Conclusion

Bio-C Temp showed alkaline pH and increased calcium ion release during the experimental period. Bio-C Temp and Ultracal XS can not be removed entirely from the canal walls after the removal protocol was employed.
